# Neuroanatomical Dissections of Unilateral Visual Neglect Symptoms: ALE Meta-Analysis of Lesion-Symptom Mapping

**DOI:** 10.3389/fnhum.2012.00230

**Published:** 2012-08-10

**Authors:** Magdalena Chechlacz, Pia Rotshtein, Glyn W. Humphreys

**Affiliations:** ^1^Department of Experimental Psychology, University of OxfordOxford, UK; ^2^School of Psychology, University of BirminghamBirmingham, UK

**Keywords:** unilateral neglect, lesion-symptom mapping, allocentric, egocentric, spatial attention

## Abstract

Unilateral visual neglect is commonly defined as impaired ability to attend to stimuli presented on the side of visual space contralateral to the brain lesion. However, behavioral analyses indicate that different neglect symptoms can dissociate. The neuroanatomy of the syndrome has been hotly debated. Some groups have argued that the syndrome is linked to posterior parietal cortex lesions, while others report damage within regions including the superior temporal gyrus, insula, and basal ganglia. Several recent neuroimaging studies provide evidence that heterogeneity in the behavioral symptoms of neglect can be matched by variations in the brain lesions, and that some of the discrepancies across earlier findings might have resulted from the use of different neuropsychological tests and/or varied measures within the same task for diagnosing neglect. In this paper, we review the evidence for dissociations between both the symptoms and the neural substrates of unilateral visual neglect, drawing on ALE (anatomic likelihood estimation) meta-analyses of lesion-symptom mapping studies. Specifically, we examine dissociations between neglect symptoms associated with impaired control of attention across space (in an egocentric frame of reference) and within objects (in an allocentric frame of reference). Results of ALE meta-analyses indicated that, while egocentric symptoms are associated with damage within perisylvian network (pre- and postcentral, supramarginal, and superior temporal gyri) and damage within sub-cortical structures, more posterior lesions including the angular, middle temporal, and middle occipital gyri are associated with allocentric symptoms. Furthermore, there was high concurrence in deficits associated with white matter lesions within long association (superior longitudinal, inferior fronto-occipital, and inferior longitudinal fasciculi) and projection (corona radiata and thalamic radiation) pathways, supporting a disconnection account of the syndrome. Using this evidence we argue that different forms of neglect link to both distinct and common patterns of gray and white matter lesions. The findings are discussed in terms of functional accounts of neglect and theoretical models based on computational studies of both normal and impaired attention functions.

## Introduction

The complexity of the visual world requires us to have the ability to select and process behaviorally relevant stimuli while ignoring the rest of the scene. We also need to have the capacity to shift attention between different elements as we search for relevant stimuli. The cognitive processes that underlie these abilities are collectively known as visuospatial attention. These cognitive mechanisms are indispensable for numerous daily activities, as illustrated by the immense problems experienced by individuals suffering from visuospatial deficits after brain damage. The most widely studied disorder of visuospatial attention is unilateral visual neglect (a lack of awareness of space contralateral to the side of brain damage; Heilman and Valenstein, 1979). In extreme cases unilateral neglect manifests itself when patients ignore food on one half of their plates or dress only half of their body. The unilateral neglect syndrome has a significant impact on daily activities and is correlated with poor recovery and return to independent living following the stroke (e.g., Campbell and Oxbury, 1976; Denes et al., 1982; Luaute et al., 2006). This disorder not only has a significant impact on the overall outcome following brain damage but also has proved to be difficult to understand and treat (e.g., Kerkhoff, 2001; Parton et al., 2004; Singh-Curry and Husain, 2010).

In the past three decades, there has been much clinical interest in understanding both cognitive symptoms and the underlying lesion anatomy of unilateral neglect. Notably, many important insights into the functional and structural organization of the neural networks involved in visuospatial attention come from neuropsychological studies examining patients with cognitive deficits associated with unilateral neglect. Specifically, these reports support notion that a distributed neuronal network of frontal and parietal areas, the fronto-parietal network, controls, and allocates visual attention (e.g., Mesulam, 1981; Corbetta and Shulman, 2002). However, the neuroanatomy of the syndrome has been hotly debated with various groups presenting different arguments for critical lesion site associated with unilateral neglect. Interestingly, the behavioral analyses indicate that unilateral neglect is a heterogeneous disorder and different neglect symptoms can dissociate, both within and across patients (e.g., Humphreys and Riddoch, 1994, 1995; Walker and Young, 1996; Doricchi and Galati, 2000; Olson, 2003). Our aim here was to provide an overall review and statistical analysis of the neuroanatomical findings, focusing on whether heterogeneity in the behavioral symptoms of neglect can be matched by variations in the brain lesions associated with different deficits. We ask whether some of the discrepancies across findings might have resulted from a failure to take into account the behavioral dissociations between patients.

The textbook diagnosis of unilateral neglect is made when patients fail to attend to stimuli presented on the side of space contralateral to their lesions (Heilman and Valenstein, 1979). However, this diagnosis does not take into account that unilateral neglect represents a complex syndrome with different patients showing a varied combination of impairments (Kerkhoff, 2001; Buxbaum et al., 2004). Although unilateral visual neglect is the most commonly diagnosed problem, the presence of neglect symptoms in different modalities has been also reported, though the prevalence varies across patients (Halligan and Marshall, 1994b; Vuilleumier et al., 1998; Kerkhoff, 2001; Hillis et al., 2005; Marsh and Hillis, 2008). Dissociations between symptoms of neglect syndrome have also been found for different sectors of space and the severity of deficits observed in individual patients depends on the magnitude and type of cognitive process affected. For example the extent of visuospatial impairments characteristic of neglect may be exacerbated by deficits in non-spatial cognitive process (Singh-Curry and Husain, 2010) and difficulty in assessment of neglect can be linked to the fact that some heterogeneity across tasks might be due to differences in (non-spatial) attentional demands (see for example Bonato et al., 2010; Bonato et al., 2012). Overall the heterogeneous deficits associated with unilateral neglect syndrome can be categorized into spatial (e.g., spatial attention, spatial bias, and visuospatial short term memory) and non-spatial (e.g., target detection, reorienting, and overall vigilance) impairments (for a recent review, see Corbetta and Shulman, 2011). Due to the variety of cognitive deficits contributing to neglect, the diagnosis of the syndrome based on any one single clinical measure may obscure the heterogeneity of symptoms. Dissociable cognitive deficits within the neglect syndrome have been previously reported both across a variety of different measures (e.g., line cancelation versus bisection) and even within the same task, perhaps depending on the way stimuli are spatially represented (Buxbaum et al., 2004; Rorden et al., 2006; Chechlacz et al., 2010; Verdon et al., 2010; Bickerton et al., 2011). Importantly, the heterogeneity in the cognitive deficits and symptoms reported in unilateral neglect patients can be matched by variations in the brain lesions associated with these different cognitive problems (Hillis et al., 2005; Mannan et al., 2005; Rorden et al., 2006; Kleinman et al., 2007; Butler et al., 2009; Malhotra et al., 2009; Medina et al., 2009; Rossit et al., 2009; Chechlacz et al., 2010; Verdon et al., 2010). This is of particular significance as it could account for the discrepancies across earlier studies using lesion-symptom mapping, which might have resulted from a failure to take into account the behavioral dissociations between patients (see also Rorden et al., 2006; Saj et al., 2012). Specifically, some groups have previously argued that the syndrome is linked to damage to the posterior parietal cortex, while others have reported damage within brain regions including the superior temporal gyrus, insula, and basal ganglia (on the one hand, see Vallar and Perani, 1986; Mort et al., 2003; Vallar et al., 2003; on the other, see Karnath, 2001; Karnath et al., 2001, 2004a). It should be noted that neglect symptoms often observed in acute stroke patients with sub-cortical lesions including the basal ganglia and thalamus (e.g., Vallar and Perani, 1986; Karnath et al., 2002) have been linked to dysfunction (abnormally perfused but structurally intact brain tissue) of cortical areas such as inferior parietal lobule and/or superior temporal gyrus (e.g., Hillis et al., 2002; Karnath et al., 2005). Thus direct contribution of the sub-cortical lesion to neglect is still debatable.

**Figure 1:**
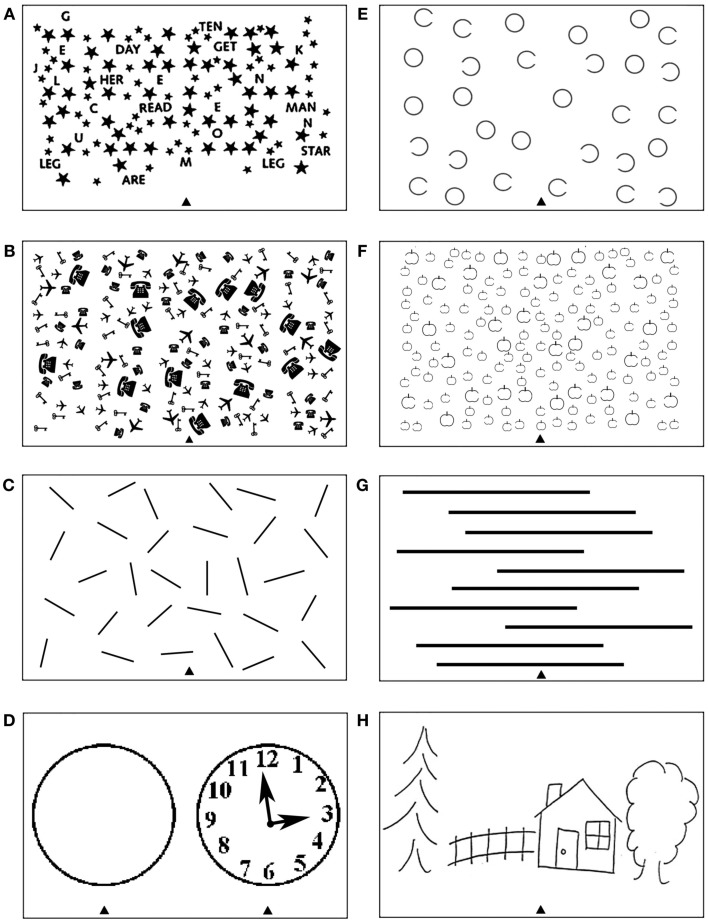
**Examples of tests frequently used to diagnose heterogeneous symptoms associated with unilateral visual neglect, which can provide measure of deficits associated with impaired control of attention either (A–D) across space, i.e., egocentric frame of reference and/or (E–H) within objects, i.e., allocentric frame of reference (see Introduction for further details)**. Common cancelation tests: **(A)** star cancelation, **(B)** key cancelation, and **(C)** line crossing, all administered by asking patients to cross targets (small stars, keys, or lines respectively) evenly distributed on the centrally placed sheet of paper – deficits are measured by target omissions on either left or right side of space. **(D)** Clock drawing test that can be administered by either asking patients to place numbers on the face of the clock or asking patients to copy the fully drawn clock (the face of the clock or fully drawn clock are centrally presented on the sheet of paper). Gap detection tests: **(E)** Ota test and **(F)** Apples Cancelation, both administered by asking patients to cross only full targets (full circles or full apples respectively) evenly distributed on the centrally placed sheet of paper – deficits are measured by counting missing targets on either left or right side of space as well as false-positive responses, i.e., crossing objects with either left or right openings). **(G)** Line bisection test, which is administered by asking patients to mark middle of a series of horizontally presented lines – deficits are measured by deviation from the center of each line. **(H)** Scene copying task, which is administered by asking patients to copy multi-object scene consisting of several elements horizontally distributed on the centrally presented sheet of paper – deficits are measured by omissions of left or right sided elements of the scene as well as omissions of either left of right side of individual elements/objects).

The most common tests used to diagnose neglect include various cancelation, line bisection, word reading, and copying scenes. Depending on their design, the tests measure deficits of spatial attention either across space in relation to the body (in an egocentric frame of reference; Riddoch and Humphreys, 1983; Doricchi and Galati, 2000) and/or across parts within objects (in an allocentric frame of reference; Walker and Young, 1996; Walker et al., 1996; Doricchi and Galati, 2000; Olson, 2003; Kleinman et al., 2007). Several different cancelation tests have been used to measure the ability to attend to stimuli presented on the right and left side of visual space (see for example Figures 1A–C). Typically such tests are administered by asking patients to cross targets evenly distributed on a centrally placed sheet of paper. In contrast to exceptions such as the line crossing test (Albert, 1973; Figure 1C), cancelation measures often require participants to select targets appearing amongst mixed sets of distractors (Mesulam, 1985; Gauthier et al., 1989; Halligan et al., 1989; Figures 1A,B). Perhaps not surprisingly, since the tests involve both target detection and selection, it has been demonstrated that these are more sensitive to mild to moderate symptoms than tasks such as line crossing (Vanier et al., 1990). Other common clinical tasks involve drawing and copying, which requires both producing elements within an egocentric frame whilst also aligning parts in their correct co-locations, perhaps using allocentric coding (e.g., either to code parts relative to a whole object or objects relative to one another; see Ishiai et al., 1993; Figure 1D; Ogden, 1985; Figure 1H). Assessments which attempt to behaviorally tease apart egocentric and allocentric symptoms include gap detection tests, such as the Ota test (Ota et al., 2001; Hillis et al., 2005; Medina et al., 2009; Figure 1E) and the Apples Cancelation test (Chechlacz et al., 2010; Bickerton et al., 2011; Humphreys et al., 2011; Figure 1F), and also word reading tests (Subbiah and Caramazza, 2000; Medina et al., 2009; Ptak et al., 2012). Gap detection tests are administered by asking patients to cross only full targets (e.g., full circles and full apples as illustrated in Figures 1E,F). Egocentric deficits are then measured by counting missing targets on either left or right side of the page while allocentric deficits are measured by counting false-positive responses (i.e., by crossing out distractors with either left or right openings; see Figures 1E,F). Finally, line bisection (Heilman and Valenstein, 1979; Figure 1G) typically involves asking patients to mark the middle of a series of horizontally presented lines. Some researchers have suggested that bisection is not a sensitive tool to detect neglect while others have debated whether bisection performance can reflects either deficits in separate coding of the ends of the lines in relation to the patient using an egocentric frame of reference, or the perception of the line as a single object in an allocentric frame of reference (Ferber and Karnath, 2001; Rorden et al., 2006; Chechlacz et al., 2010; Karnath and Rorden, 2012; see also Molenberghs and Sale, 2011 for a contrasting view).

The differences between the various diagnostic tests are of particular relevance if they underlay contrasting results on lesion-symptom mapping. Here we attempted to formally test this based on ALE meta-analyses examining whether there is a concurrence in findings dissociated based on the different neglect measure criteria. While some earlier analyses tended to assess neglect mainly in terms of line bisection tasks or deficits pooled across line bisection and cancelation (Mort et al., 2003; Mannan et al., 2005; Bird et al., 2006), other studies have diagnosed neglect using a battery of tasks which all include some degree of spatial exploration (Karnath et al., 2002, 2004a, 2009, 2011). By contrast, many recent studies following Binder et al.’s (1992) and Rorden et al.’s (2006) suggestion that different neglect symptoms may be associated with damage to discrete brain areas, have made attempts to distinguish the neuroanatomical basis of different neglect symptoms (Binder et al., 1992; Rorden et al., 2006). The emerging evidence indicates that different spatial symptoms of neglect (e.g., within allocentric and egocentric frames of reference) are associated with contrasting brain lesions (Hillis et al., 2005; Medina et al., 2009; Chechlacz et al., 2010; Verdon et al., 2010; Ptak et al., 2012; see below). For example, we have previously demonstrated that, after right hemisphere damage, left allocentric neglect is associated with lesions to the right posterior superior temporal sulcus, angular, middle temporal/inferior temporal, and middle occipital gyri, while left egocentric neglect is linked to more right anterior lesions within perisylvian network including the middle frontal, postcentral, supramarginal, and superior temporal gyri as well as the insula (Chechlacz et al., 2010). Several other research groups have reported similar dissociations (e.g., Hillis et al., 2005; Medina et al., 2009; Verdon et al., 2010; Ptak et al., 2012). Importantly, these dissociations have been noted across a variety of different tasks including gap detection and figure copy tests which can simultaneously measure both symptoms (e.g., Hillis et al., 2005; Medina et al., 2009; Chechlacz et al., 2010) as well as variety of word reading tests (e.g., Medina et al., 2009; Ptak et al., 2012).

It should also be noted that, in addition to the gray matter lesions associated with unilateral neglect, many reports have linked the symptoms of neglect to the presence of white matter lesions, which disrupt connectivity within the brain’s attentional networks. This has led some researchers to regard neglect as a disconnection syndrome (Doricchi and Tomaiuolo, 2003; Bartolomeo et al., 2007). Specifically, neglect has been reported following damage to the superior longitudinal (SLF; Doricchi and Tomaiuolo, 2003;Thiebaut de Schotten et al., 2005, 2008; He et al., 2007; Karnath et al., 2009; Shinoura et al., 2009;Chechlacz et al., 2010, 2011; Urbanski et al., 2011), the inferior longitudinal fasciculi (ILF; Bird et al., 2006; Chechlacz et al., 2010; Riddoch et al., 2010), and the inferior fronto-occipital fasciculi (IFOF; Urbanski et al., 2008, 2011; Karnath et al., 2009; Chechlacz et al., 2010; Riddoch et al., 2010).

The lesion-symptom mapping procedures used to understand the neuroanatomical basis of neglect are not un-controversial (see for example Karnath et al., 2004b versus Mort et al., 2004). Traditional lesion-symptom mapping approaches have used lesion overlap/lesion subtraction methods, contrasting lesion maps for different groups of patients categorically defined as having a particular deficit (Damasio and Damasio, 1989). More recent procedures have been developed to enable continuous behavioral measures to be used and formal statistical comparisons to be made (voxel-based lesion-symptom mapping, VLSM or voxel-wise lesion-behavior mapping, VLBM; Bates et al., 2003; Rorden et al., 2007, 2009; Karnath et al., 2009; voxel-based morphometry, VBM; Ashburner and Friston, 2000). These emerging approaches facilitate the direct quantitative examination of dissociations between the heterogeneous symptoms that contribute to disorders such as unilateral neglect (e.g., Medina et al., 2009; Chechlacz et al., 2010; Verdon et al., 2010).

In the current study, we review the evidence for dissociations between both the symptoms and the neural substrates of unilateral visual neglect, drawing on meta-analyses of published lesion-symptom mapping studies. The work evaluates the concurrence between findings from various lesion-symptom mapping studies examining the neuroanatomy of the neglect syndrome by employing coordinate based anatomic likelihood estimation (ALE) meta-analyses. Specifically, our analyses examined (i) the overall convergence between the results from different lesion-symptom mapping studies concerned specifically with visual neglect (we included here all studies that matched this criterion regardless of the assessment tools they employed), (ii) the concurrence in the damage within white matter pathways associated with neglect symptoms, and (iii) the concurrence in lesion sites associated with deficits in the control of attention either across space (egocentric frame of reference) or within objects (allocentric frame of reference), to evaluate whether behavioral dissociations within the neglect syndrome are matched by different lesion patterns. For this last analysis we fractionated data from studies using different assessment tools to measure neglect symptoms. It should be noted that our classification of the differential assessments of neglect depends on inferences about the underlying processes. For example, we assume that tasks such as line cancelation require that multiple stimuli are coded in relation to the patient’s body (e.g., using an egocentric reference frame). In contrast, tasks such as line bisection could reflect either separate coding of the perceived ends of the lines in relation to the patient (i.e., egocentric spatial coding) or perception of the line as a single object (i.e., allocentric spatial coding; Humphreys and Riddoch, 1994, 1995). Thus we examined concurrence in findings based on both line bisection and other measures of within object deficits grouped together as well as treating them as separate behavioral measures.

Based on the evidence from our meta-analyses we argue that different forms of neglect link to both distinct and common patterns of gray and white matter lesions. The results provide insights into the discrepancies that exist between different reports examining the lesion site(s) associated with unilateral visual neglect as well as providing evidence for disconnection accounts of the syndrome. The findings are discussed in terms of functional accounts of neglect and theoretical models based on computational modeling of both normal and impaired attention functions.

## Materials and Methods

### Literature search and selection criteria

For the purpose of the current study we conducted a systematic literature search to indentify relevant papers reporting the neuronal substrates of the heterogeneous symptoms associated with unilateral visual neglect. All searches were carried out using PubMed^1^ and Web of Knowledge^2^ databases. The database searches were conducted using the following keywords: (visual neglect OR unilateral neglect OR spatial neglect OR line bisection OR target cancelation) AND (anatomy OR neuroanatomy OR tractography OR diffusion tensor imaging OR perfusion weighted imaging OR diffusion weighted imaging OR lesion-symptom mapping OR VBM OR VLSM OR computed tomography OR magnetic resonance imaging). In addition, we also identified studies through references cited by review papers and through references from relevant papers found via database searches.

The inclusion criteria were as follows: (1) studies published in peer-reviewed journals; (2) use of lesion-symptom mapping approaches as defined in the Introduction, i.e., either lesion subtraction methods (based on either comparisons between the lesion overlap plots from patients with and without neglect or formal subtraction plots between the groups), VBM, or VLSM/VLBM methods; (3) the studied sample consisted of mainly brain injured patients and both experimental and control patients groups were described/defined clearly; (4) the findings were reported using spatial coordinates in either Montreal Neurological Institute (MNI; Evans et al., 1993) or Talairach space (Talairach and Tournoux, 1988); (5) papers defined neglect based on common assessment tools including at least one of the following: target cancelation, bisection, word reading, and figure copy (see Figure 1). In cases where standard coordinates were not reported in the lesion-symptom analyses, we contacted the authors to request this information. If the authors agreed to provide the information, the studies were included in our meta-analyses (we thank the following authors for providing these additional data on our request: Bird et al., 2006; Medina et al., 2009; Eschenbeck et al., 2010; Karnath et al., 2011; Vossel et al., 2011; Saj et al., 2012). We excluded studies that were (1) not published in English; (2) reported either preliminary findings or conference presentations; (3) single case studies or multiple-case studies based on patients pre-selected according to either lesion location or cognitive deficits without comparison to appropriate patient control groups (studies using traditional lesion overlap analysis based on overlapping the lesion maps of patients with certain deficit and defining an area of maximum overlap as the brain region critically sub-serving the cognitive function impaired in the patients); (4) functional neuroimaging studies (fMRI, PET, etc.) in either patients or healthy controls.

Following the literature search, we created lists of reported peak coordinates (foci) for each individual study entered into our ALE meta-analyses. In Table 1 under Analysis 1 we list the number of all foci as reported/defined by the authors of each study based on all relevant analyses of the neuroanatomy of neglect (for example if authors report both peak coordinates from lesion subtraction and VLSM, all these were listed). We entered into analysis all coordinates that were given by the authors to describe their results, i.e., all coordinates listed in text, tables, or figures excluding only these that were repeated for example both in text and tables, etc. In case of studies that do not provide a single peak coordinate but border coordinates of maximum overlap for neglect group versus controls (studies exclusively based on lesion subtraction analyses), we entered an “averaged” peak to prevent misrepresentation of data and inflating results by a number of peaks entered into analysis. In the subsequent analyses we only used the foci that were relevant to either specific types of neglect (these were selected based on reported measures of neglect as listed in Table 5) and/or specifically white matter substrates of neglect.

**Table 1:** **Studies included in the ALE meta-analyses**.

No	Study	Type of patients/time	No. of patients^‡^	Modality	Methods		No. of Foci*
					Lesion reconstruction	Data analysis*	
**ANALYSIS 1**							
1	Bird et al. (2006)	SO/AS	15	CT, MRI	M	LO/LS	1
2	Chechlacz et al. (2010)	SP/CH	38 (19)	MRI, DTI	A	VBM, VLSM, VA-FA	55
3	Chechlacz et al. (2011)	SP/CH	50	MRI	A	VBM	9
4	Chechlacz et al. (2012)	SO/AS and CH**	160	CT	A	VBM (AS and CH)	30
5	Doricchi and Tomaiuolo (2003)	SO/AS	31	CT, MRI	M	LO/LS, VLSM	12
6	Eschenbeck et al. (2010)	SO/AS	68	CT, MRI	M	VLSM	5
7	Golay et al. (2008)	SO/AS	50	CT, MRI	M	VLSM	2
8	Grimsen et al. (2008)	SO/AS + CH	21	CT, MRI	M	LO/LS	6
9	Karnath et al. (2001)	SO/AS	50	CT, MRI	M	LO/LS	4
10	Karnath et al. (2002)	SO/AS	32	CT, MRI	M	LO/LS	7
11	Karnath et al. (2004a)	SO/AS	140	CT, MRI	M	LO/LS, VLSM	2
12	Karnath et al. (2011)	SO/AS and CH**	54	CT, MRI	M	VLSM (AS and CH)	1
13	Lee et al. (2010)	SO/AS	42	SPECT	A	VLSM	12
14	Medina et al. (2009)	SO/AS	171	PWI, DWI	M	VLSM	4
15	Molenberghs and Sale (2011)	SO/AS	44	MRI	A	VLSM	2
16	Mort et al. (2003)	SO/AS	35	MRI	M	LO/LS	3
17	Ptak et al. (2012)	SO/AS	54	CT, MRI	M	LO/LS, VLSM	20
18	Rorden et al. (2006)	SO/AS	22	CT, MRI	M	LO/LS	2
19	Saj et al. (2012)	SO/AS and CH**	69	MRI	M	VSLM (AS and CH)	4
20	Urbanski et al. (2011)	SO/AS + CH	24 (12)	DTI	A	VA-FA	11
21	Verdon et al. (2010)	SO/AS	80 (41)	CT, MRI	M	LO/LS, VSLM	9
22	Vossel et al. (2011)	SO/AS	56	CT, MRI	M	VLSM	5
**ANALYSIS 2**							
1	Chechlacz et al. (2010)	SP/CH	38 (19)	MRI, DTI	A	VBM, VLSM, VA-FA	55
2	Chechlacz et al. (2011)	SP/CH	50	MRI	A	VBM	9
3	Chechlacz et al. (2012)	SO/AS and CH**	160	CT	A	VBM (AS and CH)	17
4	Grimsen et al. (2008)	SO/AS + CH	21	CT, MRI	M	LO/LS	5
5	Karnath et al. (2001)	SO/AS	50	CT, MRI	M	LO/LS	4
6	Karnath et al. (2002)	SO/AS	32	CT, MRI	M	LO/LS	7
7	Karnath et al. (2004a)	SO/AS	140	CT, MRI	M	LO/LS, VLSM	2
8	Karnath et al. (2011)	SO/AS and CH**	54	CT, MRI	M	VLSM (AS and CH)	1
9	Medina et al. (2009)	SO/AS	171	PWI, DWI	M	VLSM	1
10	Molenberghs and Sale (2011)	SO/AS	44	MRI	A	VLSM	1
11	Ptak et al. (2012)	SO/AS	54	CT, MRI	M	VLSM	4
12	Saj et al. (2012)	SO/AS and CH**	69	MRI	M	VSLM (AS and CH)	2
13	Urbanski et al. (2011)	SO/AS + CH	24 (12)	DTI	A	VA-FA	3
14	Verdon et al. (2010)	SO/AS	80	CT, MRI	M	VSLM	5
15	Vossel et al. (2011)	SO/AS	56	CT, MRI	M	VLSM	3
**ANALYSIS 3**							
1	Chechlacz et al. (2010)	SP/CH	38 (19)	MRI, DTI	A	VBM, VLSM, VA-FA	29
2	Chechlacz et al. (2012)	SO/AS and CH**	160	CT	A	VBM	17
3	Grimsen et al. (2008)	SO/AS + CH	21	CT, MRI	M	LO/LS	1
4	Lee et al. (2010)	SO/AS	42	SPECT	A	VLSM	5
5	Medina et al. (2009)	SO/AS	171	PWI, DWI	M	VLSM	3
6	Molenberghs and Sale (2011)	SO/AS	44	MRI	A	VLSM	1
7	Ptak et al. (2012)	SO/AS	54	CT, MRI	M	VLSM	11
8	Rorden et al. (2006)	SO/AS	22	CT, MRI	M	LO/LS	2
9	Verdon et al. (2010)	SO/AS	80	CT, MRI	M	VSLM	1
10	Vossel et al. (2011)	SO/AS	56	CT, MRI	M	VLSM	2
**ANALYSIS 4**							
1	Lee et al. (2010)	SO/AS	42	SPECT	A	VLSM	5
2	Molenberghs and Sale (2011)	SO/AS	44	MRI	A	VLSM	1
3	Rorden et al. (2006)	SO/AS	22	CT, MRI	M	LO/LS	2
4	Vossel et al. (2011)	SO/AS	56	CT, MRI	M	VLSM	2
**ANALYSIS 5**							
1	Chechlacz et al. (2010)	SP/CH	38 (19)	MRI, DTI	A	VBM, VLSM, VA-FA	29
2	Chechlacz et al. (2012)	SO/AS and CH**	160	CT	A	VBM	17
3	Grimsen et al. (2008)	SO/AS + CH	21	CT, MRI	M	LO/LS	1
4	Medina et al. (2009)	SO/AS	171	PWI, DWI	M	VLSM	3
5	Ptak et al. (2012)	SO/AS	54	CT, MRI	M	VLSM	11
6	Verdon et al. (2010)	SO/AS	80	CT, MRI	M	VSLM	1
**ANALYSIS 6**							
1	Chechlacz et al. (2010)	SP/CH	38 (19)	MRI, DTI	A	VBM, VLSM, VA-FA	37
2	Chechlacz et al. (2011)	SP/CH	50	MRI	A	VBM	3
3	Chechlacz et al. (2012)	SO/AS and CH	160	CT	A	VBM	12
4	Doricchi and Tomaiuolo (2003)	SO/AS	31	CT, MRI	M	LO/LS, VLSM	11
5	Golay et al. (2008)	SO/AS	50	CT, MRI	M	VLSM	1
6	Karnath et al. (2002)	SO/AS	32	CT, MRI	M	LO/LS	3
7	Mort et al. (2003)	SO/AS	35	MRI	M	LO/LS	1
8	Ptak et al. (2012)	SO/AS	54	CT, MRI	M	VLSM	6
9	Urbanski et al. (2011)	SO/AS + CH	24 (12)	DTI	A	VA-FA	11
10	Verdon et al. (2010)	SO/AS	80 (41)	CT, MRI	M	LO/LS, VSLM	5
**ANALYSIS 7**							
1	Chechlacz et al. (2010)	SP/CH	38 (19)	MRI, DTI	A	VBM, VLSM, VA-FA	26
2	Chechlacz et al. (2011)	SP/CH	50	MRI	A	VBM	3
3	Chechlacz et al. (2012)	SO/AS and CH	160	CT	A	VBM	7
4	Karnath et al. (2002)	SO/AS	32	CT, MRI	M	LO/LS	3
5	Ptak et al. (2012)	SO/AS	54	CT, MRI	M	VLSM	2
6	Urbanski et al. (2011)	SO/AS + CH	24 (12)	DTI	A	VA-FA	3
7	Verdon et al. (2010)	SO/AS	80	CT, MRI	M	VSLM	1
**ANALYSIS 8**							
1	Chechlacz et al. (2010)	SP/CH	38 (19)	MRI, DTI	A	VBM, VLSM, VA-FA	21
2	Chechlacz et al. (2012)	SO/AS and CH	160	CT	A	VBM	7
3	Ptak et al. (2012)	SO/AS	54	CT, MRI	M	VLSM	4
4	Verdon et al. (2010)	SO/AS	80	CT, MRI	M	VSLM	1

^‡^Numbers in brackets indicate that some of the included data were based on the subset of patients participating in the given study; *the information in the table only includes data analysis methods and number of foci from the identified papers that were included in the ALE meta-analysis; **these studies present separate findings for subacute and chronic phase following stroke (AS and CH) and thus the findings were included as separate experiments (see Materials and Methods); Type of patients: SO, stroke only; SP, stroke plus other brain damaged patients; Time: AS, acute and/or subacute patients; CH, chronic; AS + CH, both subacute and chronic patients were included in the same data analyses presented in the given study; Lesion reconstruction: A, automated/semi-automated; M, manual demarcation of lesion; Neuroimaging modality: CT, computed tomography; DTI, diffusion tensor imaging; DWI, diffusion weighted imaging; MRI, magnetic resonance imaging, mainly structural anatomical scan such as T1- and/or T2-weighted scans); PWI, perfusion weighted imaging; SPECT, single photon emission computed tomography; Data analyses methods: LO/LS, lesion overlap/lesion subtraction plots (please note that all included studies are based on subtraction analysis, i.e., either comparisons between the lesion overlap plots from patients with and without neglect symptoms or formal subtraction plots between patients with and without neglect symptoms; VA-FA, voxel-wise analysis of fractional anisotropy maps; VBM, voxel-based morphometry; VLSM, voxel-based lesion-symptom mapping; findings from the same paper from on separate analyses based on different methods were included as separate experiments (see Materials and Methods).

### Data analyses – design

In order to examine dissociations between both the symptoms and the neural substrates of neglect, we performed several different ALE meta-analyses (see below for Materials and Methods description). The relevant papers included in these analyses are listed in Table 1 (see also Results). *Analysis 1* included data from all relevant papers reporting the neural substrates of unilateral visual neglect considering it as a unitary syndrome, not separating out patients according to different types of symptom as well as not differentiating between gray versus white matter lesions associated with the syndrome. Specifically, in *Analysis 1* we included all the data from studies examining neglect, diagnosing neglect either from one of the commonly used tests or a battery of measures without applying any prior selection criteria. *Analyses 2* and *Analysis 3* directly examined the link between the heterogeneity of neglect and any associated neural substrates by fractionating lesion sites associated with deficits in allocating attention either across space (using an egocentric frame of reference, *Analysis 2*) or within objects (using an allocentric frame of reference, *Analysis 3*). The data included in *Analysis 2* came from studies that defined neglect exclusively using either target cancelation tests or both target cancelation and figure copying tests, measuring the patient’s ability to attend to stimuli presented on the right and left side of egocentric space (see Figure 1; Table 5). In contrast the data included in *Analysis 3* came from studies that defined neglect using a variety of tests measuring spatial deficits in relation to an allocentric frame of reference. This analysis included data from studies that employed different gap detection tests, multi-object scene copying tasks, word reading tests, and line bisection (see Figure 1; Table 5). Line bisection tests (Figure 1G) are administered by asking patients to mark the middle of horizontally presented lines and it has been suggested that the performance on this test may reflects deficits in the perception of the line as a single object (i.e., coding space within an allocentric frame of reference; Chechlacz et al., 2010; Karnath and Rorden, 2012). However, despite the fact that neglect symptoms measured using line bisection can dissociate from these measured by target cancelation (Ferber and Karnath, 2001; Rorden et al., 2006; for an opposite view however, see Molenberghs and Sale, 2011), it has been suggested that bisection deficits can reflect problems in coding of the perceived ends of the lines in relation to the patient (i.e., within an egocentric frame of reference); for example, it has been observed that the magnitude of any asymmetries in bisection increase when the lines are presented further to the contralesional side of a patient’s body (Riddoch and Humphreys, 1983). Consequently we also examined the concurrence in lesion sites associated with poor performance on line bisection test only (*Analysis 4*) followed by analysis of concurrence in lesion sites associated more specifically with deficits in the control of attention within objects (in an allocentric frame of reference) after excluding reports using line bisection (*Analysis 5*). As recently much attention has been given to white matter lesions and disconnection accounts of the syndrome, *Analysis 6* was performed on a subset of data specifically describing the link between neglect symptoms and white matter damage based on data from identified studies diagnosing neglect based on either one of the commonly used tests or on battery of neglect diagnostic measures without applying any selection criteria. Finally, *Analyses 7* and *Analysis 8* examined the link between white matter lesions and the specific symptoms of neglect associated with the control of attention either in relation the patient’s body (egocentric deficits, *Analysis 7*) or within objects (allocentric deficits, *Analysis 8*).

### ALE meta-analyses

We performed the meta-analyses using BrainMap GingerALE 2.1 software^3^ to estimate the concurrence between the reported neuroanatomy of unilateral visual neglect from different published studies and to examine the evidence for dissociations between neglect symptoms and the underlying neural substrates of the syndrome. The inputs for the different analyses were as defined above and all source papers are listed in Table 1. We performed all analyses in MNI space and if necessary we converted coordinated reported by authors in Talairach space to MNI space using the coordinate conversion tool implemented in GingerALE software.

Traditionally GingerALE is used for activation likelihood estimation (ALE) meta-analyses using as inputs coordinates describing foci identified in functional neuroimaging studies (Turkeltaub et al., 2002; Laird et al., 2005). However, GingerALE also performs anatomic likelihood (ALE) meta-analyses (Ellison-Wright et al., 2008; Glahn et al., 2008; Di et al., 2009; Ferreira et al., 2011) that assess the overlap between anatomical foci identified by different research groups using voxel-wise analyses of structural neuroimaging data, as for example here the foci obtained based on various lesion-symptom mapping approaches. In the current paper we applied the revised version of the ALE method (Eickhoff et al., 2009) after implementing the modified ALE algorithm design to minimize within-experiment and within-group effects (Turkeltaub et al., 2012). The ALE algorithm of Turkeltaub et al. (2012) was used here to control for dependent within-group effects as some of the papers included here report findings based on different data analysis approaches (e.g., lesion overlap and VLSM) or included data based on cognitive measures obtained in the same group of patients but at two separate time points (e.g., in the subacute and chronic phases following stroke) and these were input as separate coordinates lists (i.e., separate experiments; see Table 1). The ALE approach models the anatomical foci from different published reports (here studies listed in Table 1) as Gaussian probability density distribution at a given coordinate. First Gaussian widths are calculated based on the expected between-template variability in spatial normalization and the relationship between the sample size and inter-subject localization uncertainty. Next for each individual experiment (here referring to single analysis reported in each lesion-symptom mapping paper), a Modelled Activation Map (MA map) is calculated by taking the voxel-wise union of the Gaussians for all the foci reported by that specific experiment (Eickhoff et al., 2009). Following that, an ALE map (experimental ALE map) is generated as the voxel-wise union of all MA maps from the full datasets (all included experiments from published studies). To differentiate true concurrence of foci from random clustering (random spatial associations), the calculated experimental ALE map is tested against ALE null distribution maps generated by permutation test to represent the same number of foci as the real analysis but randomly redistributed throughout the brain. In the current study we used a statistical threshold of *p* < 0.05 FDR (False Discovery Rate) corrected for multiple comparisons and a minimum cluster size of 200 mm^3^ (Eickhoff et al., 2009). ALE maps were overlaid onto the MNI template using MRIcron software MRICro (Chris Rorden, McCausland Center for Brain Imaging, University of South Carolina, SC, USA). The anatomical localization of the significant clusters identified by the meta-analyses was based on the Duvernoy Human Brain Atlas (Duvernoy et al., 1991), the Woolsey Brain Atlas by (Woolsey et al., 2008), and the Mori MRI Atlas of Human White Matter (Mori, 2005).

## Results

Table 1 presents a list and details of all the reviewed studies that fulfilled the inclusion criteria as specified in the Section “Materials and Methods” and were included in ALE meta-analyses. All studies that were included in the ALE meta-analyses presented below reported the neuronal substrates of left unilateral visual neglect. Twenty-two studies (1306 participants; total of 32 experiments with 238 relevant foci identified) that met the inclusion criteria were identified and their data entered into *Analysis 1* (overall concurrence in the reported neural substrates of unilateral visual neglect; not applying any selection criteria with regards to the tests of neglect).

For *Analysis 2* we identified 15 studies (1043; total of 20 experiments with 149 relevant foci identified; Table 1) that met the selection criteria and for *Analysis 3* we included ten studies (688 participants; total of 13 experiments with 75 relevant foci identified; Table 1) examining concurrence in the neuronal substrates associated with egocentric and allocentric neglect respectively. We included four studies (164 participants; total of 4 experiments with 13 relevant foci identified; Table 1) reporting the neural substrates associated with asymmetric line bisection (*Analysis 4*) and six studies (524 participants; total of 9 experiments with 62 relevant foci; Table 1) reporting the neural substrates associated with deficits in the control of attention within objects – in this case using measures excluding line bisection (*Analysis 5*). Ten of the identified studies (554 participants; total of 16 experiments with 101 relevant foci identified) reported neglect associated with damage within white matter (not applying any selection criteria with regards to the tests of neglect) and these data were included in *Analysis 6* (Table 1). Seven of these studies (438 participants; total of 11 experiments with 54 relevant foci identified; Table 1) specifically reported white matter lesions associated with egocentric symptoms (included in *Analysis 7*) and four studies (332 participants; total of 7 experiments with 33 relevant foci; Table 1) specifically reported white matter lesions associated with allocentric neglect (these were included in *Analysis 8*).

### Neural substrates of unilateral visual neglect syndrome – Analysis 1

**Figure 2:**
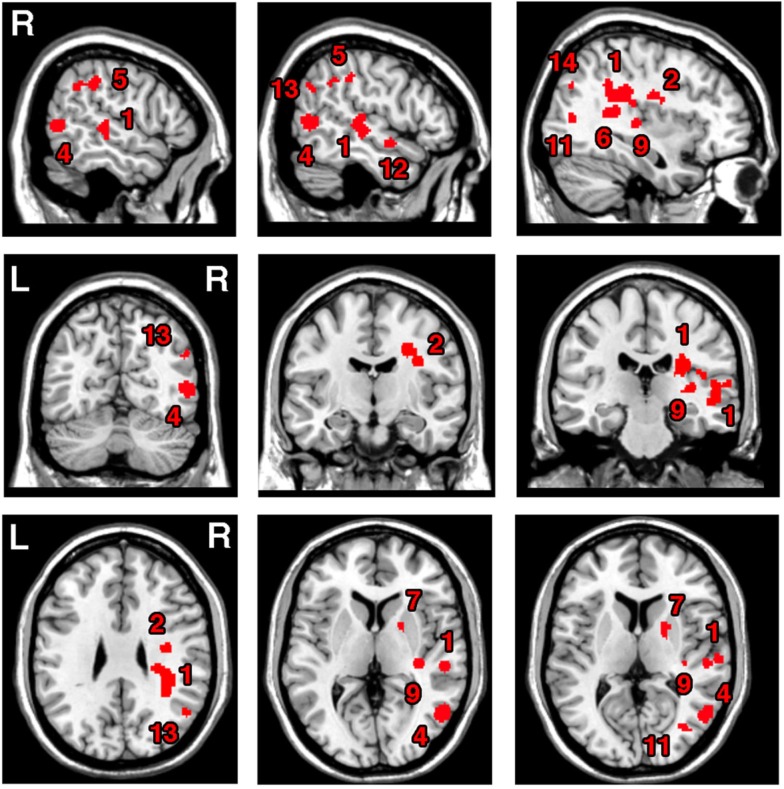
**Significant clusters identified in *Analysis 1* (ALE meta-analysis, *p* < 0.05, FDR corrected for multiple comparisons, cluster size >200 mm^3^) – the convergence between results from different lesion-symptom mapping studies concerned with neuroanatomy of the unilateral visual neglect syndrome**. The numbers denote indentified ALE clusters as listed in Table 2.

The ALE meta-analysis for the main effect, i.e., the overall concurrence in the reported neural substrates of unilateral visual neglect (not differentiating between either different symptoms or gray versus white matter lesions) revealed 15 significant clusters (Table 2; Figure 2). However, we found that the agreement between different studies was not very strong with only one cluster showing high convergence with 16 out of 32 experiments contributing and 4 other clusters with 5 or more contributing experiments. The most concurrent cluster was located sub-cortically within long association pathways including the SLF (ALE peaks at MNI 36, −26, 26 and 22, −30, 24) and superior thalamic radiation (ALE peak at MNI 30, −24, 22). Part of this cluster also covered some areas of right cerebral cortex including the superior temporal gyrus (BA 22, ALE peak at MNI 54, −28, 2) and the inferior parietal lobule (BA 40, ALE peak at MNI 34, −46, 34) and extending into TPJ (BA 21/22/39, ALE peak at MNI 50, −38, 18). The four other clusters were located in the right insula (BA 13, ALE peak at MNI 36, −12, 26), the middle temporal gyrus (BA 21 54, −64), the postcentral gyrus (BA2, ALE peak at MNI 26, −40, 52), and the inferior parietal lobule (both supramarginal and angular gyrus/BA 40 and BA 39) extending into TPJ (BA 40/22, ALE peak at MNI 56, −34, 38).

**Table 2:** **Significant ALE clusters and corresponding MNI coordinates identified in Analysis 1**.

Cluster			ALE value	MNI coordinates			No. of Exp.*
No	Anatomical label	Size (mm^3^)		*X*	*Y*	*Z*	
1	Right SLF	5784	0.026	36	−36	26	16
	Right SLF, superior thalamic radiation		0.02	30	−24	22	
	Right SLF		0.017	22	−30	24	
	Right inferior parietal lobule (IPL)/BA40		0.016	34	−46	34	
	Right superior temporal gyrus/BA 22		0.021	54	−28	2	
	Right superior temporal gyrus		0.016	50	−22	−4	
	Right superior temporal gyrus		0.015	48	−24	14	
	Right superior temporal gyrus		0.016	60	18	8	
	Right superior temporal gyrus		0.013	44	−34	20	
	Right lateral fissure, TPJ junction BA 21/22/39		0.013	50	−38	18	
2	Right IFOF, superior corona radiata	1776	0.025	26	−10	36	7
	Right insula/BA 13		0.018	36	−12	26	
	Right SLF		0.014	20	0	34	
	Right insula/BA 13		0.013	36	−6	22	
3	Right postcentral/BA 2 and supramarginal gyrus/BA 40	1464	0.031	26	−40	52	7
4	Right middle temporal gyrus/BA 21	1312	0.03	54	−64	4	6
5	Right supramarginal gyrus/BA 40, TPJ BA 40/22	816	0.017	56	−34	38	5
	Right angular gyrus/BA 39		0.016	54	−48	34	
6	Right IFOF	664	0.018	36	−46	12	4
	Right posterior thalamic radiation		0.018	36	−42	14	
7	Right putamen	544	0.017	22	4	8	4
8	Right putamen	400	0.019	20	8	−10	3
9	Right ILF, IFOF	368	0.018	34	−26	4	3
10	Right precuneus/BA 7	336	0.019	8	−38	18	2
11	Right middle occipital gyrus/BA 19	320	0.016	34	−74	8	2
12	Right superior temporal gyrus/BA 22	304	0.016	52	−2	−12	3
13	Right angular gyrus/BA 39	240	0.016	50	−62	30	3
14	Right middle occipital gyrus/BA 19	232	0.015	38	−76	34	2
15	Right inferior occipital/lingual BA 18	200	0.015	26	−86	−8	2

*Number of contributing experiments (No, number); BA, Brodmann Area; IFOF, inferior fronto-occipital fasciculus; ILF, inferior longitudinal fasciculus; SLF, superior longitudinal fasciculus; TPJ, temporo-parietal junction.

### Dissociating the neural substrates of egocentric and allocentric neglect – Analyses 2–5

**Figure 3:**
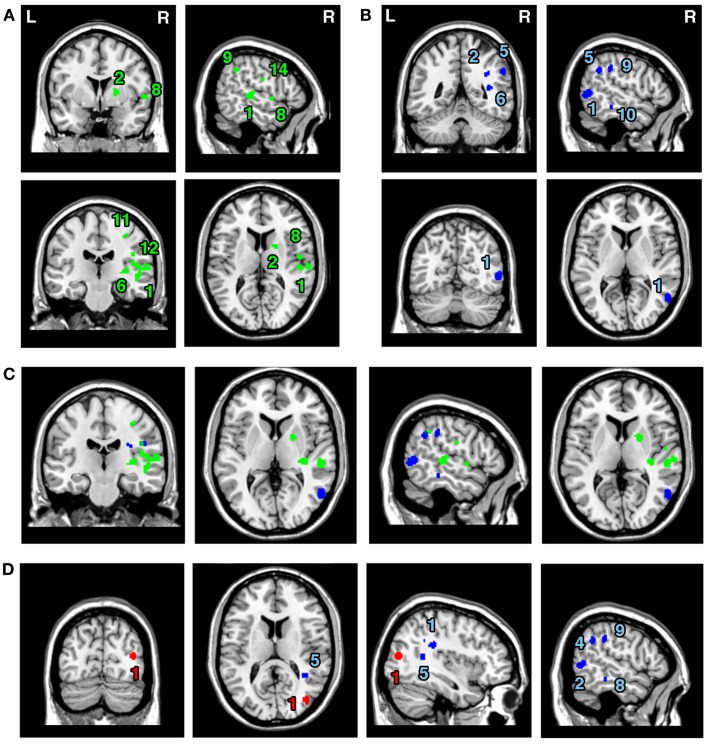
**Significant clusters identified in *Analyses 2*, *3*, *4*, and *5* (ALE meta-analyses, *p* < 0.05, FDR corrected for multiple comparisons, cluster size >200 mm^3^)**. The concurrence in lesion sites associated with impaired control of attention either **(A)** across space, i.e., egocentric frame of reference (green; *Analysis 2*) or **(B)** within objects, i.e., allocentric frame of reference (blue; *Analysis 3*) including lesion sites associated with impaired performance on line bisection test. **(C)** Distribution of ALE clusters indentified in both analyses (*Analysis 2* in green and *Analysis 3* in blue). **(D)** The concurrence in lesions associated with either impaired performance on line bisection (red; *Analysis 4*) or impaired control of attention within objects, i.e., allocentric frame of reference as measured by various neglect diagnostic tests excluding line bisection (*Analysis 5*; blue). The numbers in **(A,B,D)** denote indentified ALE clusters as listed in Table 3.

The ALE meta-analyses examining concurrence in the lesion sites associated with the control of attention either in relation to the patient’s body or within objects (in egocentric or allocentric frames of reference) revealed 16 significant clusters associated with egocentric symptoms and 10 clusters associated with allocentric symptoms (Table 3; Figures 3A,B). The convergence between studies included in both *Analysis 2* and *Analysis 3* was not as robust as in the case of the white matter analysis reported below but the findings were nevertheless striking. The two most concurrent clusters (contributed respectively by 7 and 5 out of 20 experiments) identified in *Analysis 2* (egocentric neglect) were located within the right superior temporal gyrus (BA 22, ALE peak at MNI 54, −28, 2), right insula (BA 13, ALE peak at MNI 46, −22, 14), and sub-cortical structures including the right putamen (ALE peak at MNI 20, 4, 8) and thalamus (ALE peak at MNI 22, −2, 0). The two most concurrent clusters (contributed respectively by 4 and 6 out of total of 13 experiments) identified in *Analysis 3* (allocentric neglect) were located within the right angular gyrus (BA39, ALE peak at MNI 42, −48 30), right temporo-parietal junction (BA 22/39, ALE peak at MNI 44, −34, 22), right middle temporal gyrus (BA 21, ALE peak at MNI 54, −64, 4), and sub-cortically within the right SLF (ALE peak at MNI 36, −36, 28). Strikingly, these two analyses indicated that, while egocentric symptoms were associated with damage within perisylvian network (the pre- and postcentral, supramarginal, and superior temporal gyri) and damage within sub-cortical structures, more posterior lesions including the angular, middle temporal, and middle occipital gyri were associated with allocentric symptoms (Figure 3C).

**Table 3:** **Significant ALE clusters and corresponding MNI coordinates identified in Analyses 2, 3, 4, and 5**.

Cluster			ALE value	MNI coordinates			No. of Exp.*
No	Anatomical label	Size (mm^3^)		*X*	*Y*	*Z*	
**ANALYSIS 2**							
1	Right superior temporal gyrus/BA22	2160	0.02	54	−28	2	7
	Right superior temporal gyrus		0.017	62	−22	8	
	Right insula/BA13		0.014	46	−22	14	
	Right superior temporal gyrus/BA22		0.014	50	−20	−2	
	Right insula/BA13		0.013	44	−14	0	
2	Right putamen	1360	0.018	20	4	8	5
	Right putamen, thalamus		0.012	22	−6	−2	
	Right thalamus		0.013	22	−2	0	
3	Right supramarginal gyrus/BA40	640	0.018	24	−42	52	3
4	Right caudate	464	0.016	24	−30	24	3
5	Right putamen	576	0.019	20	8	−10	3
6	Right ILF, IFOF	552	0.017	34	−24	4	3
7	Right precentral gyrus/BA4	344	0.015	42	−8	60	2
8	Right insula/BA13	216	0.014	50	−10	10	2
9	Right supramarginal gyrus/BA40	240	0.014	54	−46	36	2
10	Right superior temporal gyrus/BA22	232	0.014	58	2	0	2
11	Right precentral gyrus/BA4, SLF	272	0.012	34	−24	50	3
12	Right SLF	240	0.014	44	−22	26	2
13	Right precentral gyrus/BA4	224	0.014	32	−14	50	2
14	Right postcentral gyrus/BA2/3	216	0.013	54	−12	26	2
**ANALYSIS 3**							
1	Right middle temporal gyrus/BA21/BA37	816	0.02	54	−64	4	4
2	Right SLF	912	0.015	36	−36	28	6
	Right lateral fissure, TPJ/BA21/22/39		0.012	44	−34	22	
	Right angular gyrus/BA39		0.012	42	−48	30	
3	Right middle occipital gyrus/BA19	372	0.013	40	−76	12	2
4	Right intraparietal sulcus/BA2/3	384	0.014	50	−20	26	2
5	Right angular gyrus, TPJ/BA39/22/40	344	0.016	54	−48	34	2
6	Right IFOF	312	0.016	36	−48	12	2
7	Right SLF	304	0.012	28	−24	28	2
8	Right superior parietal lobule/BA5	304	0.014	22	−44	50	2
9	Right intraparietal sulcus, TPJ/BA40/22	208	0.012	56	−34	36	2
10	Right inferior temporal gyrus/BA20	208	0.012	58	−32	−14	2
**ANALYSIS 4**							
1	Right middle occipital gyrus/BA19 (extending into superior temporal sulcus)	278	0.012	40	−78	14	2
**ANALYSIS 5**							
1	Right SLF	952	0.015	36	−36	28	6
	Right lateral fissure, TPJ/BA22/39		0.012	44	−34	22	
	Right angular gyrus/BA39		0.012	42	−48	30	
2	Right middle temporal gyrus/BA21	600	0.019	54	−62	4	3
3	Right intraparietal sulcus/BA2/3	384	0.013	50	−20	26	2
4	Right angular gyrus, TPJ/BA39/22/40	368	0.016	54	−48	34	2
5	Right IFOF	312	0.016	36	−48	12	2
6	Right SLF	312	0.012	28	−24	28	2
7	Right superior parietal lobule/BA5	304	0.014	22	−44	50	2
8	Right inferior temporal gyrus/BA20	208	0.012	58	−32	−14	2
9	Right intraparietal sulcus, TPJ/BA40/22	208	0.012	56	−34	36	2

*Number of contributing experiments (No, number); BA, Brodmann Area; IFOF, inferior fronto-occipital fasciculus; ILF, inferior longitudinal fasciculus; SLF, superior longitudinal fasciculus; TPJ, temporo-parietal junction.

We next examined whether there was a difference in the neural substrates associated with neglect symptoms defined by poor line bisection performance (*Analysis 4*) and the neural substrates associated with allocentric symptoms as measured by diagnostic tests excluding line bisection (*Analysis 5*). The ALE meta-analysis on biases in line bisection revealed one significant cluster (Table 3; Figure 3D) located within the right temporo-occipital junction, the right middle occipital gyrus extending into the superior temporal sulcus (BA 19, ALE peak at MNI 40, −78, 14). However, it should be noted that this finding was based on a comparison across only four papers (total of 4 experiments with total of only 13 foci). In contrast to this, after excluding the data based on line bisection, we found strong concurrence in the reported damage associated with allocentric symptoms with six out of nine experiments contributing to the largest of the identified clusters (Table 3). This cluster indicated high convergence of reported lesions in the right hemisphere within the right angular gyrus (BA 39, ALE peak at MNI 42, −48, 30), the right TPJ (BA 22/39, ALE peak at MNI 44, −34, 22), and the right SLF (ALE peak at MNI 36, −36, 28).

### White matter substrates of visual neglect – Analyses 6–8

**Figure 4:**
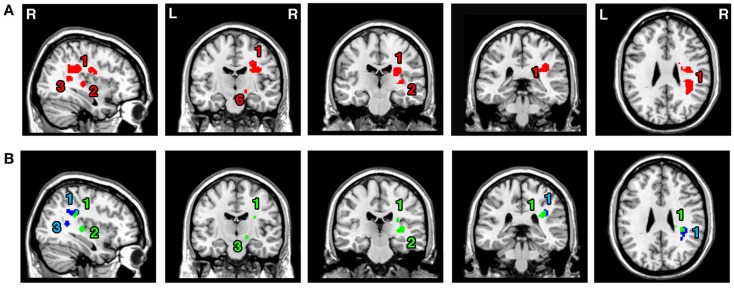
**Significant clusters within the white matter identified in *Analyses 6*, *7*, and *8* (ALE meta-analysis, *p* < 0.05, FDR corrected for multiple comparisons, cluster size >200 mm^3^)**. **(A)** The convergence between results from different lesion-symptom mapping studies examining link between damage within white matter pathways and unilateral visual neglect syndrome (*Analysis 6*). **(B)** The concurrence in white matter lesions associated with impaired control of attention either across space, i.e., egocentric frame of reference (*Analysis 7*; green) or within objects, i.e., allocentric frame of reference (*Analysis 8*; blue). The numbers denote indentified ALE clusters as listed in Table 4.

The ALE meta-analysis examining concurrence in the reported damage within white matter pathways associated with left neglect, not differentiating between the different symptoms of neglect (*Analysis 6*), revealed six significant clusters (Table 4; Figure 4A) located within the long association and projection pathways including the SLF, IFOF, ILF, corona radiata, thalamic radiation, and internal capsule in the right hemisphere. Strikingly, we found high concurrence between the studies with 14 out of 16 experiments contributing to the largest of the identified clusters (cluster 1; Table 4; Figure 4A). This cluster covered two of the long association pathways – the SLF (ALE peaks at MNI 36, −36, 26 extending till 36, −6, 22) and the IFOF (ALE peak at MNI 26, −10, 36 and 32, −54, 32) as well as superior parts of the corona radiata (ALE peak at 26, −10, 36) and the thalamic radiation (ALE peak at MNI 28, −22, 22). Subsequent, ALE meta-analyses examining concurrence in white matter lesions associated with the control of attention across either egocentric space (*Analysis 7*) or allocentric space (*Analysis 8*) revealed four significant clusters associated with egocentric symptoms and four clusters with allocentric symptoms (Table 4; Figure 4B). Overall, we found higher concurrence between studies reporting white matter damage associated with allocentric symptoms compared to that between studies reporting white matter damage associated with egocentric symptoms. The most concurrent cluster (contributed respectively by 5 out of total of 11 experiments) identified in *Analysis 7* examining neural substrates of left egocentric symptoms was located within right SLF (ALE peak at MNI 34, −34, 26), right superior thalamic radiation (ALE peak at MNI 30, −20, 24), and right ILF (ALE peak at 30, −28, 24). The most concurrent cluster (contributed to respectively by five out of seven experiments in *Analysis 8*) was located within the right SLF (ALE peak at MNI 36, −34, 30), the right IFOF, and the right posterior thalamic radiation (ALE peak at MNI 32, −48, 30). Strikingly, these two analyses (*Analyses 7* and *8*) indicated that egocentric and allocentric symptoms were associated with lesions within common long association and projection pathways, in particular the SLF and the thalamic radiation.

**Table 4:** **Significant ALE clusters located within white matter and corresponding MNI coordinates identified in Analyses 6, 7, and 8**.

Cluster			ALE value	MNI coordinates			No. of Exp.*
No.	Anatomical label	Size (mm^3^)		*X*	*Y*	*Z*	
**ANALYSIS 6**							
1	Right SLF	8416	0.026	36	−36	26	14
	Right superior corona radiata, IFOF		0.025	26	−10	36	
	Right superior thalamic radiation		0.024	28	−22	22	
	Right SLF		0.018	36	−12	26	
	Right superior thalamic radiation, SLF		0.017	22	−30	22	
	Right SLF		0.014	20	0	34	
	Right SLF		0.014	36	−6	22	
	Right IFOF		0.011	32	−54	32	
2	Right ILF, IFOF	616	0.018	34	−26	4	3
3	Right IFOF	488	0.017	36	−48	12	3
4	Right posterior thalamic radiation	384	0.014	22	−42	50	3
5	Right internal capsule (posterior limb)	256	0.012	16	−14	−8	2
**ANALYSIS 7**							
1	Right superior thalamic radiation, SLF	1520	0.016	24	−28	24	5
	Right SLF		0.013	34	−36	26	
	Right superior thalamic radiation		0.011	30	−20	24	
	Right SLF, ILF		0.011	30	−28	24	
	Right SLF		0.011	28	−12	24	
2	Right ILF, IFOF	640	0.017	34	−24	4	3
3	Right internal capsule (posterior limb)	368	0.011	16	−14	−8	2
4	Right posterior thalamic radiation	360	0.014	22	−44	50	2
**ANALYSIS 8**							
1	Right SLF	1184	0.021	36	−34	30	5
	Right IFOF, posterior thalamic radiation		0.012	32	−48	30	
2	Right posterior thalamic radiation	544	0.014	22	−44	50	3
	Right IFOF, posterior thalamic radiation		0.011	26	−40	44	
3	Right IFOF	352	0.016	36	−48	12	2
4	Right SLF, anterior thalamic radiation	224	0.012	22	0	36	2

*Number of contributing experiments (No, number); IFOF, inferior fronto-occipital fasciculus; ILF, inferior longitudinal fasciculus; SLF, superior longitudinal fasciculus.

**Table 5:** **Neglect measures and reported symptoms in studies included in ALE meta-analyses**.

No	Study	Neglect measure(s)	Reported symptoms
1	Bird et al. (2006)	Battery of different measures including BIT* plus other cancelation, bisection, and drawing tests	Overall neglect (across various measures)
2	Chechlacz et al. (2010)	Apples cancelation test**	Allocentric, egocentric
3	Chechlacz et al. (2011)	Apples cancelation test**	Allocentric, egocentric
4	Chechlacz et al. (2012)	Apples cancelation test**	Allocentric, egocentric
5	Doricchi and Tomaiuolo (2003)	Cancelation and line bisection tests	Overall neglect (across various measures)
6	Eschenbeck et al. (2010)	Behavioral inattention test (BIT)*, daily living activities assessment	Overall neglect (across various measures)
7	Golay et al. (2008)	Battery of different measures including cancelation, line bisection, and drawing tests	Overall neglect (across various measures)
8	Grimsen et al. (2008)	Battery of different measures based on BIT* plus search paradigms	Allocentric (across different measures), egocentric (across different measures)
9	Karnath et al. (2001)	Battery of different cancelation and copying tests plus baking tray task	Egocentric (across different measures)
10	Karnath et al. (2002)	Battery of different cancelation and copying tests plus baking tray task	Egocentric (across different measures)
11	Karnath et al. (2004a)	Battery of different cancelation and copying tests plus baking tray task	Egocentric (across different measures)
12	Karnath et al. (2011)	Battery of different cancelation and copying tests	Egocentric (across different measures)
13	Lee et al. (2010)	Battery of different measures including cancelation, line and letter bisection, scene, and figure copying tests	Overall neglect (across different tests), allocentric (line bisection bias)
14	Medina et al. (2009)	Battery of different measures including cancelation, line bisection, gap detection scene copy, clock drawing, word reading tests	Allocentric (across different tests not including line bissection), egocentric (across different tests)
15	Molenberghs and Sale (2011)	Cancelation and line bisection tests	Allocentric (line bisection bias), egocentric (cancelation laterality)
16	Mort et al. (2003)	Cancelation and line bisection tests	Overall neglect (across different tests),
17	Ptak et al. (2012)	Battery of different measures including cancelation, line bisection, and scene copy tests plus tests for neglect dyslexia	Overall neglect (across different tests), allocentric (object-centered reading errors), egocentric (page-centered errors)
18	Rorden et al. (2006)	Battery of different measures including cancelation, line bisection, scene copy, and clock drawing tests	Allocentric (line bisection bias)
19	Saj et al. (2012)	Battery of different measures including cancelation, line bisection, scene copy, clock drawing, writing, and text reading tests	Overall neglect (across different tests), egocentric (across cancelation and copying tests)
20	Urbanski et al. (2011)	Battery of different measures including cancelation, line bisection, scene copy, and overlapping figures tests	Overall neglect (across different tests), egocentric (cancelation laterality)
21	Verdon et al. (2010)	Battery of different measures including cancelation, line bisection, scene copy, gap detection, word, and text reading tests	Overall neglect (across different tests), allocentric, egocentric, visuo-motor
22	Vossel et al. (2011)	Behavioral inattention test (BIT)*	Allocentric (line bisection bias), egocentric (overall cancelation laterality)

*BIT, battery of tests including line bisection, line and star cancelation, copying of figures, text reading, and clock drawing (Wilson et al., 1987b); **type of gap detection test (Bickerton et al., 2011).

## Discussion

Here, we examined data indicating dissociations between the heterogeneous symptoms and the neural substrates of unilateral visual neglect based on ALE meta-analyses of lesion-symptom mapping studies. There is a substantial body of evidence demonstrating that different neuropsychological tests and/or even varied measures within the same task used for diagnosing neglect can reveal different symptoms of this heterogeneous syndrome whilst also varying in their overall sensitivity for detecting mild to moderate symptoms (e.g., Vanier et al., 1990; Ferber and Karnath, 2001; Rorden et al., 2006; Bickerton et al., 2011). Past studies have hotly disputed the neuroanatomy of unilateral neglect, while, in contrast, more recent studies have suggested that at least some of the previously reported differences between studies can stem from the heterogeneity of the syndrome and the associated lesion sites. In the current paper, we provide statistical evidence supporting this notion based on the ALE meta-analyses. We first examined whether there was commonality across studies when the different tests of neglect are not taken into account. In this overall assessment (*Analysis 1*), the consistency across the reported findings was relatively poor, though one of the identified clusters was contributed to by approximately 50% of all experiments. This covered regions within posterior parietal cortex (IPL), the insula, and the thalamus as well as within white matter pathways. Strikingly, when the different tests were not differentiated, there was high overall concurrence in white matter lesions within the long association SLF (inferior fronto-occipital and ILF) and projection (corona radiata and thalamic radiation) pathways. This provides strong evidence for a disconnection account of the syndrome, which can generate a common pattern of deficit across different tests. While the assessment of common cortical damage across the different tests of neglect generated moderate results, the results were stronger when we separated out tests sensitive to the positions of elements in egocentric and allocentric reference frames. Here our concurrence analyses indicated that egocentric symptoms were associated with damage within the perisylvian network (the pre- and postcentral, supramarginal, and superior temporal gyri) along with damage within sub-cortical structures, while more posterior lesions including the angular, middle temporal, and middle occipital gyri) were associated with allocentric symptoms.

### Unilateral visual neglect – the controversial quest for a key lesion site

Understanding lesion-symptom relations in patients with cognitive deficits has both clinical and basic scientific implications. Data from individuals who have impaired cognitive processes due to the specific patterns of neural damage allow researchers to shape and test theories of how the human brain works and is organized. Importantly, information about the extent and location of any lesion, and the associated cognitive problems, also carry direct implications for clinical care – specifically if predictions of outcome and plans for rehabilitation can be informed by lesion data. Unilateral visual neglect has been extensively studied by both basic scientists and clinicians as, on the one hand, the syndrome provides a unique opportunity to study human visuospatial attention, while on the other neglect-related problems have proved difficult to understand and treat. Not surprisingly, there have been numerous research efforts toward understanding the lesion patterns associated with neglect but not without controversies (e.g., see Mort et al., 2003 versus Karnath et al., 2001; Karnath et al., 2004a). Some groups have argued that the syndrome is linked to posterior parietal cortex lesions, while others report damage within regions including the superior temporal gyrus, insula, and basal ganglia (Vallar and Perani, 1986; Mort et al., 2003; Vallar et al., 2003; on the other, see Karnath, 2001; Karnath et al., 2001, 2004a). Interestingly, this debate has not only centered on the critical lesion site itself but also on the methods used to determine the link between site of brain damage and the behavioral symptoms and on patient selection criteria and assessment (e.g., Rorden et al., 2006; Medina et al., 2009; Chechlacz et al., 2010; Verdon et al., 2010). We provide here evidence across studies for dissociations between both the symptoms and the neural substrates of unilateral visual neglect, illustrated by both low and high concurrence in our ALE meta-analyses. The results presented here support the notion that the tests used to diagnose neglect symptoms are critical when studying the neuronal substrates of this heterogeneous syndrome, since the correlations between the brain lesions vary according to the cognitive process assessed in different tasks. The process rather than the test *per se* seems important and our analyses indicate that common lesion-symptom mapping occurs across different tasks, which “tap” the same process. Our conclusion is that the quest for identifying a key lesion site for unilateral neglect is an impossible task as this heterogeneous syndrome itself is not a “theoretically coherent but rather meaningless entity” (Halligan and Marshall, 1992). Our study points to the coherent evidence indicating that behavioral dissociations between particular neglect symptoms are closely coupled with anatomical dissociations and thus it seems more appropriate to define separately the key lesion site for allocentric neglect and separately for egocentric neglect, etc.

The symptoms associated with neglect are traditionally diagnosed with a battery of tests including target cancelation, line bisection and scene/figure copying (Wilson et al., 1987a,b). Additionally, gap detection and single word or sentence/paragraph reading task can be used (Subbiah and Caramazza, 2000; Ota et al., 2001; Bickerton et al., 2011). Karnath and colleagues suggested that while cancelation tests provide a good measure of core deficits associated with neglect (including biases in gaze direction, exploration, and cancelation), other diagnostic tools measure deficits behaviorally distinct from these symptoms (Ferber and Karnath, 2001; Rorden et al., 2006; Karnath and Rorden, 2012). Specifically, it has been demonstrated that line bisection bias and allocentric spatial coding, as measured on gap detection tasks, multi-object scene copying, and single word reading, can differentiate anatomically allocentric symptoms from egocentric symptoms with substantial concurrence across studies using different methods (Rorden et al., 2006; Medina et al., 2009; Chechlacz et al., 2010; Verdon et al., 2010; Ptak et al., 2012). Strikingly, our ALE analyses confirmed previously reports indicating that egocentric symptoms are associated with the damage to more anterior cortical regions, while allocentric symptoms are associated with more posterior lesions. To conclude, though there may not be a key lesion site, there are different key lesion sites according to the forms of spatial representation mediating performance.

### White matter lesions – unilateral visual neglect as a disconnection syndrome

The data presented here also provide strong evidence linking white matter disconnections to neglect. Previously there have been arguments that neglect can be viewed as a disconnection syndrome, following a simple idea that neglect symptoms result from structural disruption of connectivity within fronto-parietal attention networks (Doricchi and Tomaiuolo, 2003; Bartolomeo et al., 2007). Consistent with this, there is now a growing body of evidence that neglect is associated with damage to the SLF (Doricchi and Tomaiuolo, 2003; Thiebaut de Schotten et al., 2005, 2008; He et al., 2007; Karnath et al., 2009; Shinoura et al., 2009; Chechlacz et al., 2010, 2011), the ILF (Bird et al., 2006; Chechlacz et al., 2010; Riddoch et al., 2010), and the IFOF (Urbanski et al., 2008, 2011; Karnath et al., 2009; Chechlacz et al., 2010; Riddoch et al., 2010), i.e., the long association pathways associated with spatial attention, spatial orienting, visual selection, and spatial working memory (Aralasmak et al., 2006; Schmahmann and Pandya, 2006; Schmahmann et al., 2007). We examined here the existing evidence linking neglect symptoms with white matter lesions across different lesion-symptom mapping studies. We found convergent lesion patterns across all studies without applying any selection criteria based on the type of test used to diagnose neglect, covering both allocentric and egocentric symptoms. The high concurrence in the reported white matter lesions was found within long association (SLF, IFOF, ILF) as well as projection (corona radiata and thalamic radiation) pathways. There is a consensus on the cortical areas connected by the SLF (Petrides and Pandya, 1984, 2002; Makris et al., 2005; Schmahmann and Pandya, 2006; Schmahmann et al., 2007; Thiebaut de Schotten et al., 2012) and ILF (Catani et al., 2003; Schmahmann and Pandya, 2006) and their anatomy conserved between the monkey and the human brain. By contrast the anatomy of IFOF is somewhat controversial. Some post-mortem dissections and tractography reconstructions indicate the existence of IFOF, a white matter pathway providing direct connection between frontal and occipital lobes in the human brain (Crosby, 1962; Catani et al., 2002; Thiebaut de Schotten et al., 2012). However, since IFOF is not present in the monkey brain and since there is a documented poor correspondence between cytoarchitectonic probabilistic post-mortem histology and *in vivo* tractography based reconstructions of IFOF (Burgel et al., 1999; Thiebaut de Schotten et al., 2011), the anatomy of IFOF remains questionable. Interestingly, recent study examining the comparative anatomy of the long association pathways (including IFOF) in the rhesus monkey and human brain, has demonstrated that the anterior fibers of the extreme capsule in the monkey brain overlap with those of the human IFOF and project to similar frontal regions. On the other hand, the posterior fibers differ in human and monkey brain – in the monkey brain the posterior projections do not reach the occipital lobe and project to the temporal lobe, while human IFOF projects to the occipital lobe (Thiebaut de Schotten et al., 2012).

The concept of a “disconnection syndrome” can be traced back to the forefathers of cognitive neuropsychology such as Carl Wernicke, Hugo Liepman, and Jules Dejerine. However, the popularity of the concept can be credited to the work of Geschwind who presented a revised disconnection account of many neurological disorders (Geschwind, 1965a,b; for review, see also Catani and Ffytche, 2005; Catani and Mesulam, 2008). According to the classical disconnection concept as put forward for example by Wernicke, a disconnection syndrome can be viewed as a disorder of higher cognitive function resulting from a breakdown of associative connections between cortical areas due to white matter lesions (Wernicke, 1874). In contrast to this, Geschwind viewed disconnection syndromes as disorders of higher cognitive functions resulting from either white matter lesions or lesions within association cortices, which serve as relay posts between primary motor, primary sensory, and limbic cortical areas (Geschwind, 1965a). Regardless of the specifics of the disconnection concept, it has a very appealing applicability to syndrome of unilateral neglect and here we provide evidence supporting this notion. First, it can be argued that the cognitive processes underlying spatial attention and visual selection are derived from a widely distributed neuronal network subserved by long association fronto-parietal and fronto-occipital white matter pathways (Makris et al., 2005; Petrides and Pandya, 2006; Schmahmann and Pandya, 2006). This is in accordance with arguments such as those made by Corbetta and Shulman (2011), that neglect is better explained by the dysfunctions of distributed neuronal networks rather than by specific cortical damage. Secondly, many previous reports have demonstrated a strong relationship between white matter lesions and neglect, fitting our meta-analyses. The interesting point about our analyses, though, is that neglect symptoms which fractionate in terms of their cortical underpinning, can be linked back to common white matter damage. We consider this point below.

### Functional accounts of unilateral visual neglect

Our ALE meta-analyses supports the argument that distinct cortical regions control attention across egocentric space and within objects (“between” and “within object” spatial representations; see Humphreys, 1998). An alternative account is that egocentric neglect reflects a problem in global space perception while allocentric neglect reflects a problem in representing space at a more local scale. Halligan and Marshall (1994a) proposed that left neglect after right hemisphere damage is brought about by the combination of poor global space perception along with a spatial bias in attention. Poor attention to local spatial areas is associated with left rather than right hemisphere damage (Delis et al., 1983) and, if coupled to a spatial bias in selection, then there may be poor detection of missing parts on one side of individual objects – allocentric symptoms. However we found no evidence for this (please note that while some lesion-symptom mapping studies only included patients with right hemisphere lesions, others applied no such selection) and there was certainly no evidence that allocentric neglect was particularly associated with left hemisphere damage, as might be expected on this account. Another possibility is that both forms of neglect stem from a gradient of attention across egocentric space (e.g., Driver and Pouget, 2000). On this gradient account, there will be a bias against elements on one side of objects, even when the objects fall in the ipsilesional visual field. Again, this account has problems with the data. For example, it predicts that allocentric and egocentric neglect should co-occur behaviorally and they should be associated with common lesion sites. In contrast to this the behavioral data accumulated by various research groups (e.g., Medina et al., 2009; Chechlacz et al., 2010; Ptak et al., 2012) indicate dissociations between patients with one or other form of neglect and, in addition, egocentric, and allocentric neglect are associated with contrasting lesions. This gradient account also fails to explain prior results where opposite egocentric and allocentric biases have occurred even in the same patient, which also arose in some cases in the present sample (Humphreys and Riddoch, 1994, 1995).

The evidence supporting anatomical and behavioral dissociations between egocentric and allocentric symptoms is in agreement with computational modeling of visual attention (Heinke and Humphreys, 2003). It can be proposed that the different neural regions support the allocation of attention to the distinct spatial representations held in other areas, or the regions may support processes that read-in visual information (egocentric symptoms) or that read-out information (allocentric symptoms) from neural networks involved in selecting between stimuli as they compete for object recognition. One framework was proposed by Heinke and Humphreys (2003). In their model visual information is fed-into a selection network where separate objects compete for entry into a focus-of-attention, and activity within the focus-of-attention gates access to stored object knowledge, which is translation invariant across the retina. Selected objects are subsequently registered in a location map reflecting the salience of stimuli in the visual field (the Selective Attention for Identification Model, SAIM). Subsequently, Heinke and Humphreys demonstrated that damage affecting the visual information coming into one side of the competition network led to egocentric neglect, with there being poor recovery of stimuli on one side of retinally defined space. In contrast, damage affecting the output from the selection network going into one side of the focus-of-attention led to allocentric neglect, with the contralesional parts of objects being neglected irrespective of their lateral position in the field (Heinke and Humphreys, 2003). This argument, for distinct spatial codes being derived for different computational reasons in object processing, fits with the data on lesion dissociation that we report. Note though that common communication pathways might be set up from these different representations to output systems for motor responses, so that damage to the communication pathways leads to problems within both egocentric and allocentric space.

### Methodological Caveats

We employed here an approach based on ALE meta-analysis that traditionally is applied to data from functional neuroimaging studies and uses coordinates describing brain activation foci (GingerALE, Turkeltaub et al., 2002; Laird et al., 2005). However, GingerALE also performs anatomic likelihood (ALE) meta-analysis and in the past this method has been successfully used to assess the overlap between anatomical foci identified by voxel-wise analyses of structural neuroimaging data (Ellison-Wright et al., 2008; Glahn et al., 2008; Di et al., 2009; Ferreira et al., 2011). As the method uses as input coordinates corresponding to the statistical peak from the lesion-symptom mapping analysis, it could be argued that such points poorly represent the usually large lesions associated with neglect symptoms. This is even more problematic when using peak coordinates representing the results from lesion subtraction analyses (based on either comparisons between the lesion overlap plots from patients with and without neglect symptoms or from formal subtraction plots between patients with and without neglect symptoms), as such methods describe the areas where the groups differ quantitatively and not necessarily statistically. Furthermore, many early lesion subtraction papers differ in terms of their definition of the critical area(s) associated with neglect. While some authors provide peak coordinates of lesion overlap, others provide border coordinates of maximum overlap for neglect group versus controls. As many early influential reports examining the neuroanatomy of neglect are based on lesion subtraction methods and not statistical VLSM analyses, despite the methodological problems with peak coordinates definition, we included all such studies in the meta-analysis in order to have a full representation of published findings. Furthermore, these arguments and methodological caveats should be weighted against the fact that the ALE approach is based not on simple point plotting but on estimations of probability (see Materials and Methods for details) followed by statistical analyses corrected for the observation of false positives. Furthermore, this approach allows investigators to factor differences in the methods and sample sizes that are used by different research groups, and it appears to provide a useful way to gain an overview across lesion-symptom data.

### Conclusion

In conclusion, we argued here that different symptoms of unilateral visual neglect link to both distinct patterns of gray matter lesions and common patterns of white matter lesions. We provide here statistical evidence based on ALE meta-analyses (e.g., low overall concurrence between different studies and higher concurrence after fractionating neglect symptoms) that multiple factors arising from variability in the neglect diagnosis explain the discrepancies reported in the literature on the neuronal substrates of unilateral visual neglect. It is plausible that other modulating factors, such as for example the neuroimaging modality used (we combined here findings based on CT, anatomical MRI, DTI, and PWI), the data analysis methods (we combined here findings based on different lesion-symptom mapping approaches including both simple lesion overlap/subtraction methods as well as methods based on statistical analysis, e.g., VBM and VLSM) and the time of assessment, i.e., acute versus chronic brain injury, may also be important. However, because of the much higher concurrence demonstrated after fractionating the symptoms of neglect, we believe that the diagnostic tools are adequate to explain the literature discrepancies.

## Conflict of Interest Statement

The authors declare that the research was conducted in the absence of any commercial or financial relationships that could be construed as a potential conflict of interest.

## Footnotes

^1^http://www.ncbi.nlm.nih.gov/pubmed

^2^http://apps.webofknowledge.com

^3^http://brainmap.org/
